# Association of serum markers with improvement in clinical response measures after treatment with golimumab in patients with active rheumatoid arthritis despite receiving methotrexate: results from the GO-FORWARD study

**DOI:** 10.1186/ar3188

**Published:** 2010-11-17

**Authors:** Sudha Visvanathan, Mahboob U Rahman, Edward Keystone, Mark Genovese, Lars Klareskog, Elizabeth Hsia, Michael Mack, Jacqui Buchanan, Michael Elashoff, Carrie Wagner

**Affiliations:** 1 Centocor Research and Development, Inc., 200 Great Valley Parkway, Malvern, PA 19355, USA; 2 University of Pennsylvania School of Medicine, 3620 Hamilton Walk, Philadelphia, PA 19104, USA; 3 University of Toronto, 27 King's College Circle, Toronto, ON M5 S 3G4, Canada; 4 Mount Sinai Hospital, 600 University Avenue Toronto, ON M5G 1X5, Canada; 5 Stanford University, 450 Serra Mall, Palo Alto, CA 94305, USA; 6 Karolinska Institute, SE-171 77 Stockholm, Sweden and Karolinska Hospital, Huddinge 141 86 Stockholm, Sweden; 7 Johnson & Johnson Pharmaceutical Services, LLC, 200 Great Valley Parkway, Malvern, PA 19355, USA; 8 Elashoff Consulting, 11124 Yellow Leaf Way, Germantown, MD 20876, USA; 9 Current address: Hoffman La-Roche, 340 Kingsland Street, Nutley, NJ 07110, USA; 10 Current address: Pfizer Inc., 500 Arcola Road, Collegeville, PA 19426, USA; 11 Current address: Buchanan Biotech Consulting, PO Box 1326, Mountain View, CA 94042, USA

## Abstract

**Introduction:**

The goal of this study was to identify serum markers that are modulated by treatment with golimumab with or without methotrexate (MTX) and are associated with clinical response.

**Methods:**

Sera were collected at weeks 0 and 4 from a total of 336 patients (training dataset, *n *= 100; test dataset, *n *= 236) from the GO-FORWARD study of patients with active rheumatoid arthritis despite MTX. Patients were randomly assigned to receive placebo plus MTX; golimumab, 100 mg plus placebo; golimumab, 50 mg plus MTX; or golimumab, 100 mg plus MTX. Subcutaneous injections were administered every 4 weeks. Samples were tested for select inflammatory, bone, and cartilage markers and for protein profiling using multianalyte profiles.

**Results:**

Treatment with golimumab with or without MTX resulted in significant decreases in a variety of serum proteins at week 4 as compared with placebo plus MTX. The American College of Rheumatology (ACR) 20, ACR 50, and Disease Activity Score (DAS) 28 responders showed a distinct biomarker profile compared with nonresponding patients.

**Conclusions:**

ACR 20 and ACR 50 responders among the golimumab/golimumab + MTX-treated patients had a distinct change from baseline to week 4 in serum protein profile as compared with nonresponders. Some of these changed markers were also associated with multiple clinical response measures and improvement in outcome measures in golimumab/golimumab + MTX-treated patients. Although the positive and negative predictive values of the panel of markers were modest, they were stronger than C-reactive protein alone in predicting clinical response to golimumab.

**Trial registration:**

http://ClinicalTrials.gov identification number: NCT00264550.

## Introduction

Rheumatoid arthritis (RA) is characterized by the presence of proinflammatory cytokines, tissue-destructive enzymes, and bone degradation products in the blood, synovium, and joints. The success of antitumor necrosis factor α (anti-TNF-α) therapies in controlling RA indicates that TNF-α is a key controlling factor in driving inflammation and associated bone degradation. Several markers are known to be related to disease progression in RA (C-reactive protein (CRP), erythrocyte sedimentation rate (ESR), anti-cyclic citrullinated peptide (anti-CCP) antibodies, rheumatoid factor, and osteoprotegrin-receptor activator of nuclear factor (NF)- κB ligand) [1-3], but better clinical response markers are needed to assist rheumatologists in selecting treatments most likely to benefit any particular patient. Several studies have shown that reductions in CRP [4-7] and anti-CCP antibodies as well as rheumatoid factor [5,8,9] are associated with improvements in clinical response in patients treated with anti-TNF-α therapies. Baseline levels of intracellular adhesion molecule-1 (ICAM-1) and cartilage oligomeric matrix protein (COMP) have been associated with response in RA patients treated with adalimumab [6]. More recent studies have identified that apolipoprotein A1 [10], serpin, and S-100-related proteins are associated with response to infliximab treatment [11]. We also recently showed that changes in E-selectin, interleukin (IL)-18, serum amyloid A, and matrix metalloproteinase-9 (MMP-9) are associated with improvement in clinical response measures in a phase 2 study of patients with active RA despite methotrexate (MTX) therapy, who were treated with golimumab (a human monoclonal antibody to TNF-α) [12]. Overall, these studies included small numbers of patients and limited datasets, making it difficult to test the reproducibility or predictive power of these preliminary results; however, several of these studies showed weak associations (*r *values or odds ratios) between the identified biomarkers and specific clinical response measures.

In the current study, our primary objective was to evaluate approximately 100 different serum proteins by using multiplex and single-plex assay platforms (enzyme-linked immunosorbent assay (ELISA) and Luminex) to identify markers modulated by golimumab treatment in patients with RA. The secondary objective was to determine whether any of these markers is strongly associated with multiple clinical measures in response to golimumab. Our last objective was to evaluate whether the preliminary test results could be confirmed in a larger set of patients from the same study.

## Materials and methods

The details of the GO-FORWARD study have been previously published [13]. In brief, patients with active RA despite MTX were randomly assigned in a 3:3:2:2 ratio to receive placebo plus MTX (group 1); golimumab, 100 mg plus placebo (group 2); golimumab, 50 mg plus MTX (group 3); or golimumab, 100 mg plus MTX (group 4). At week 16, patients in groups 1, 2, or 3 who had less than 20% improvement from baseline in tender and swollen joints entered early escape. Patients in group 1 received golimumab, 50 mg, while continuing MTX; patients in group 2 received MTX while continuing golimumab, 100 mg; and patients in group 3 had their golimumab dose increased from 50 to 100 mg while continuing MTX. Patients who were originally assigned to group 4 were not eligible for treatment adjustment.

As reported previously [13], this study was conducted in accordance with the Declaration of Helsinki and good clinical practices. The protocol was reviewed and approved by each site's institutional review board or ethics committee. All patients provided written informed consent before undergoing study-related procedures.

Sites were randomly chosen for biomarker testing. Biomarker analysis was conducted on an initial subset of 100 consecutively enrolled patients from the GO-FORWARD study (hereafter referred to as the "training" subset). Samples from an additional 236 consecutively enrolled patients assigned to golimumab plus placebo and golimumab plus MTX groups (hereafter referred to as the "test" subset) from this same study were subsequently analyzed to evaluate the reproducibility of the training set results. Patient sera were collected at weeks 0, 4, 14, and 24. Samples were tested for selected markers by using Luminex and ELISA platforms by Quintiles Laboratories (Marietta, GA) and Pacific Biometrics (Seattle, WA). The individual markers selected for these analyses included bone alkaline phosphatase, COL 2-3/4C long neoepitope, deoxypyridinoline, hyaluronic acid, IL-6, IL-8, ICAM-1, MMP-3, N-terminal propeptide of type 1 procollagen (P1NP), osteocalcin, pyridinoline, TNF-α, and vascular endothelial growth factor (VEGF). The samples also were analyzed by Rules Based Medicine (Austin, TX) using the HumanMAP version 1.6 protein-profiling analysis [14]. The HumanMAP profiling analysis included 92 analytes. Some of the selected markers listed above were also included in this profile analysis (IL-6, IL-8, ICAM-1, MMP-3, TNF-α, and VEGF).

Only markers for which 20% or more of samples were above the lower limit of quantification were included in the subsequent data analysis. Biomarker data were log_2 _transformed. Changes from baseline were tested by using one-sample *t *tests. Pearson correlation was used to measure the association between biomarker levels and clinical response. Logistic regression models were used to test for the association between biomarker levels and clinical response measures and patient reported outcomes. Clinical response was evaluated by using the American College of Rheumatology response criteria (ACR 20 and ACR 50) and the Disease Activity Score using 28 joints (DAS 28). Health-related quality of life was evaluated using the 36-question Short Form Survey (SF-36). Fatigue was evaluated using the Functional Assessment of Chronic Illness Therapy-Fatigue (FACIT-F). Prediction models were developed by using logistic regression. Model accuracy (sensitivity, specificity, negative predictive value (NPV), and positive predictive value (PPV)) was estimated by using cross validation.

To account for multiple testing, a false discovery rate (FDR) analysis was performed. The FDR analysis was used to define a *P*-value threshold at which the FDR would be approximately 5% to 10% and it accounted for the fact that the biomarkers studied were not independent but showed marker-to-marker correlations.

## Results

Of the 107 biomarkers evaluated, 78 (73%) met the prespecified criteria for inclusion in the data analysis (that is, 20% or more of all samples were above the lower limit of quantification for the assay). As discussed in more detail later, we found significant relations to efficacy for biomarkers in the following general categories: acute phase reactants (α_1_-antitrypsin, CRP, haptoglobin, serum amyloid P, von Willebrand factor), bone metabolism factors (hyaluronic acid, pyridinoline, P1NP), coagulation factors (lipoprotein(a), plasminogen activator inhibitor-1 (PAI-1), factor VII), hematologic factors (complement 3, ferritin, myoglobin), inflammatory markers (CD40, ENRAGE (S100A12), epithelium-derived neutrophil-activating protein 78 (ENA-78), IL-1 receptor agonist, IL-6, IL-16, ICAM-1, macrophage inflammatory protein (MIP)-1α, MIP-1β, MMP-3, monocyte chemotactic protein-1 (MCP-1), monocyte/macrophage-derived chemokine (MDC or CCR-4), myeloperoxidase, tissue inhibitor of metalloproteinases-1 (TIMP-1), TNF receptor 2, VEGF), metabolic factors (adiponectin, apolipoprotein A1, apolipoprotein C3, leptin), and other factors (thyroxine-binding globulin, basic fibroblast growth factor (bFGF), carcinoembryonic antigen, stem cell factor, insulin, cancer antigen 125, serum glutamic oxaloacetic transaminase (SGOT), sex hormone-binding globulin (SHBG)).

### Baseline characteristics

Baseline characteristics for the training and test subsets are displayed in Table 1. The test subset was generally similar to the training subset, although the golimumab 50 mg plus MTX group in the training subset had a higher proportion of women than the other treatment groups in the training subset.

**Table 1:** Baseline characteristics for training and test datasets

	Placebo + MTX	Golimumab 100 mg + placebo	Golimumab 50 mg + MTX	Golimumab 100 mg + MTX	Total
**Training dataset**					
Number	21	30	21	28	100
Age (years)	50 ± 12 (24-76)	50 ± 12 (22-71)	53 ± 11 (25-68)	52 ± 9 (38-76)	51 ± 11 (22-76)
Weight (kg)	70 ± 13 (47-97)	72 ± 15 (47-104)	69 ± 18 (43-108)	75 ± 22 (47-120)	72 ± 17 (43-120)
Sex (% men)	10%	20%	10%	21%	16%
Race (% Caucasian)	76%	67%	67%	75%	71%
CRP (μg/ml)	1.97 ± 2.54 (0.3-10.8)	2.16 ± 2.77 (0.3-11.7)	1.20 ± 1.53 (0.3-7.0)	1.38 ± 1.44 (0.3-6.2)	1.70 ± 2.18 (0.30-11.7)
Swollen joint count	13.0 ± 5.7 (5-26)	13.9 ± 10.4 (5-51)	12.5 ± 9.2 (4-48)	14.2 ± 9.6 (5-43)	13.5 ± 9.0 (4-51)
Tender joint count	21.3 ± 12.3 (6-62)	21.4 ± 13.1 (5-58)	23.2 ± 16.8 (4-63)	24.1 ± 13.4 (6-53)	22.5 ± 13.7 (4-63)
FACIT-F (0-52)		26 ± 11 (4-50)	26 ± 10 (4-50)	27 ± 12 (12-50)	26 ± 11 (4-50)
SF-36 mental component summary score (0-100)		44 ± 9 (20-61)	45 ± 11 (26-61)	44 ± 11 (24-62)	44 ± 10 (20-62)
SF-36 physical component summary score (0-100)		30 ± 8 (17-54)	32 ± 9 (18-51)	33 ± 8 (19-52)	31 ± 8 (17-54)
					
**Test dataset**					
Number	N/A	102	68	66	236
Age (years)		50 ± 11 (21-74)	50 ± 11 (18-79)	50 ± 10 (23-72)	50 ± 11 (18-79)
Weight (kg)		74 ± 17 (42-135)	74 ± 18 (39-146)	71 ± 17 (40-136)	73 ± 17 (39-146)
Sex (% men)		22%	22%	21%	22%
Race (% Caucasian)		82%	76%	82%	81%
CRP (μg/ml)		1.84 ± 2.27 (0.3-15.1)	2.23 ± 2.54 (0.3-11.5)	1.98 ± 2.68 (0.3-16.8)	1.99 ± 2.47 (0.3-16.8)
Swollen/tender joint count		41.0 ± 21.6 (10-88)	47.4 ± 23.0 (10-105)	41.7 ± 21.0 (9-100)	43.0 ± 21.9 (9-105)
Swollen joint count		15.0 ± 10.6 (4-59)	18.0 ± 12.3 (4-53)	14.8 ± 9.7 (4-45)	15.8 ± 10.9 (4-59)
Tender joint count		26.0 ± 15.9 (5-68)	29.3 ± 15.3 (5-68)	27.0 ± 15.0 (4-62)	27.2 ± 15.5 (4-68)
FACIT-F (0-52)		29 ± 11 (5-50)	27 ± 11 (6-50)	26 ± 10 (6-47)	27 ± 11 (5-50)
SF-36 mental component summary score (0-100)		44 ± 12 (19-73)	44 ± 11 (19-73)	43 ± 12 (17-68)	43 ± 11 (17-73)
SF-36 physical component summary score (0-100)		31 ± 9 (15-54)	30 ± 8 (16-49)	29 ± 8 (12-46)	30 ± 8 (12-54)

Values are expressed as mean ± standard deviation (range), unless otherwise indicated. CRP, C-reactive protein; FACIT-F, Functional Assessment of Chronic Illness Therapy-Fatigue; MTX, methotrexate; SF-36, 36-question Short Form Survey.

In the test subset, mean baseline marker levels were similar among the treatment groups, with the exception of levels of myeloperoxidase, thyroxine-binding globulin, vascular cellular adhesion molecule-1, and TNF-α (data not shown). In the training subset, differences among the treatment groups were observed in mean myeloperoxidase and prostatic acid phosphatase levels only (data not shown). These treatment-group differences did not affect the results of the subsequent analyses. Additionally, biomarker levels were generally similar between responders and nonresponders at baseline (data not shown).

### Changes from baseline in biomarker levels

**Figure 1:**
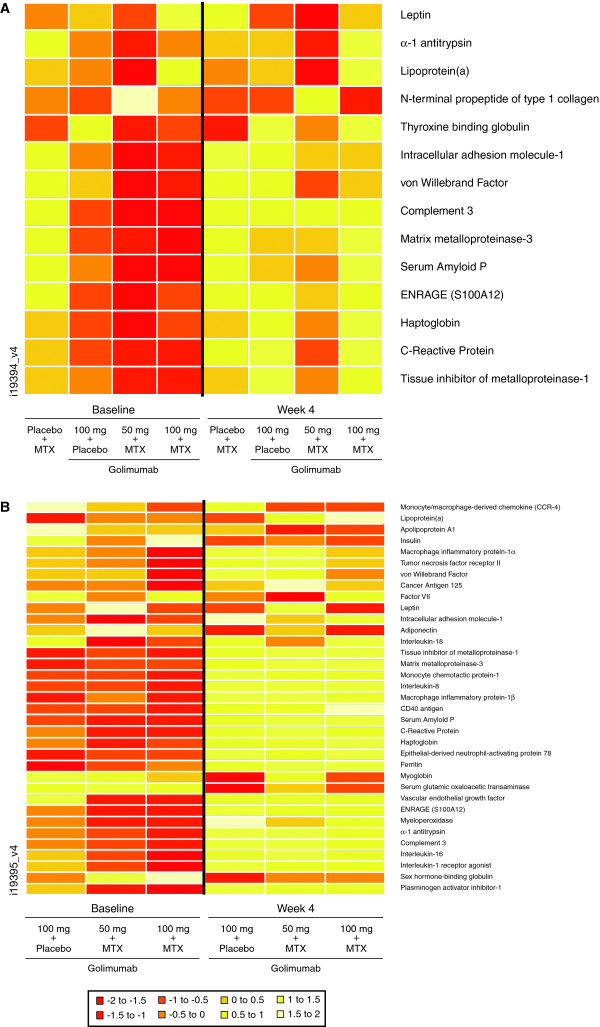
**Biomarker heatmaps for significant differences between baseline and week 4 for test and training datasets**. Heatmaps representing biomarkers that were significantly different between baseline and week 4 for any of the treatment group for the training **(a) **and test **(b) **datasets. In the test dataset, only patients treated with golimumab were evaluated. Colors represent ranges of mean log_2 _transformed biomarker levels at each time point (see legend for ranges).

In the training dataset, significantly greater decreases from baseline to week 4 (*P *< 0.01) in the mean levels of 14 markers as well as an increase in P1NP were observed in the golimumab plus MTX groups compared with the placebo plus MTX group. Log_2 _transformed values for these markers at baseline and week 4 are shown in Figure 1a. Markers with significant changes included a metabolic factor (leptin), acute-phase reactants (α_1_-antitrypsin, von Willebrand factor, serum amyloid P, haptoglobin, and CRP), a coagulation factor (lipoprotein(a)), a bone-metabolism factor (P1NP), inflammatory markers (ICAM-1, MMP-3, ENRAGE, and TIMP-1), a hematologic factor (complement 3), and thyroxine-binding globulin.

In the test dataset, a larger set of markers significantly changed after 4 weeks (Figure 1b). In addition to the markers identified earlier in the training dataset, changes were observed in inflammatory markers (MDC, MIP-1α, TNF receptor 2, IL-18, MCP-1, IL-8, MIP-1β, CD40, ENA 78, VEGF, myeloperoxidase, IL-16, IL-1 receptor agonist), coagulation factors (lipoprotein(a), factor VII, PAI-1), metabolic factors (apolipoprotein A1, adiponectin), hematologic factors (ferritin, myoglobin), and other factors (insulin, cancer antigen 125, SGOT, and SHBG). In both datasets, less substantial changes in these markers were observed in the golimumab monotherapy treatment group as compared with the golimumab plus MTX groups, indicating a stronger modulation of the overall biomarker response for golimumab treatment in combination with MTX compared with golimumab monotherapy.

**Figure 2:**
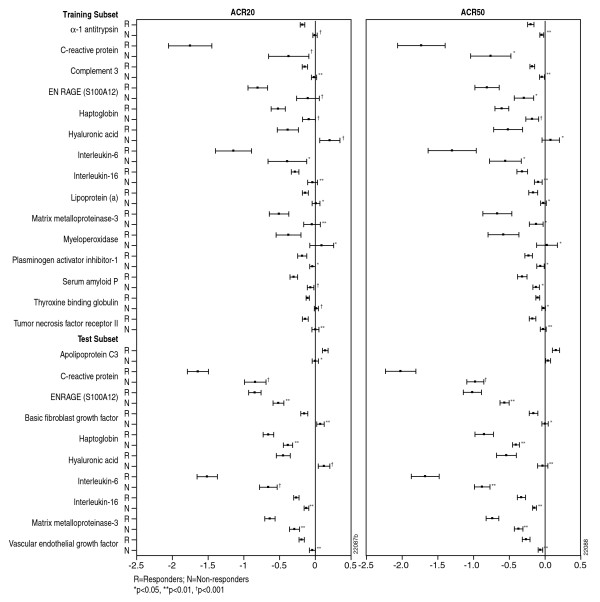
**Comparison of biomarkers significantly different between ACR responders and nonresponders for golimumab-treated patients**. Mean ± SD comparison of biomarkers (log^2 ^transformed) that were significantly different between ACR 20 and ACR 50 responders and nonresponders for golimumab-treated patients in the Training and Test Subsets.

Distinct changes in biomarker profiles were observed for golimumab-treated patients who were ACR 20 responders and nonresponders at week 14 (Figure 2). In the training dataset, ACR 20 responders had significantly greater decreases from baseline to week 4 in 16 markers compared with nonresponders. Significant differences between responders and nonresponders also were found in the test dataset for seven of these markers. Apolipoprotein C3, bFGF, and VEGF levels were the only markers for which significant differences were observed between ACR 20 responders and nonresponders in the test dataset but not in the training dataset (Figure 2). Similar markers were modulated between ACR 20 and ACR 50 responders and nonresponders.

### Associations between biomarker levels and clinical endpoints in golimumab/golimumab plus MTX-treated patients

Associations (odds ratio values) between biomarker levels and several clinical endpoints are summarized in Table 2. In the training dataset, only baseline levels of two markers (pyridinoline and von Willebrand factor) were significantly associated with selected clinical response measures in golimumab-treated patients. Baseline von Willebrand factor levels were associated with ACR 20 and ACR 50 responses at week 14, whereas baseline levels of pyridinoline were associated with ACR 20 responses only at week 14. Changes from baseline to week 4 in selected markers (including α_1_-antitrypsin, complement 3, ENRAGE, haptoglobin, hyaluronic acid, IL-8, IL-16, MMP-3, pyridinoline, PAI-1, serum amyloid P, and thyroxine-binding globulin) were also associated with clinical response measures at week 14.

**Table 2:** Associations between biomarker levels (baseline and changes from baseline to week 4) and clinical measures (ACR 20, ACR 50, and DAS 28 responses) at week 14 for golimumab/golimumab + MTX training and test datasets

	ACR 20		ACR 50		DAS 28	
	
	OR	*P*	OR	*P*	OR	*P*
**Training subset**						
*Baseline*						
Pyridinoline	0.101	0.004	0.383	NS	0.150	NS
von Willebrand factor	0.444	0.048	0.366	0.026	0.420	NS
*Change from baseline to week 4*						
_α1_-Antitrypsin	0.025	0.009	1.021	NS	0.138	NS
Complement 3	0.007	0.006	0.707	NS	0.324	NS
ENRAGE (S100A12)	0.455	0.015	0.841	NS	0.381	0.030
Haptoglobin	0.131	0.003	0.693	NS	0.276	0.033
Hyaluronic acid	0.343	0.006	0.975	NS	0.511	NS
Interleukin-8	0.483	NS	0.472	0.020	0.572	NS
Interleukin-16	0.121	0.012	1.041	NS	0.096	0.015
Matrix metalloproteinase-3	0.318	0.008	1.102	NS	0.283	NS
Plasminogen activator-1	0.114	0.041	0.713	NS	0.442	NS
Pyridinoline	3.744	NS	0.212	0.044	23.610	NS
Serum amyloid P	0.057	0.006	0.650	NS	0.075	0.035
Thyroxine-binding globulin	0.008	0.008	2.093	NS	0.221	NS
						
**Test subset**						
*Baseline*						
Apolipoprotein C3	0.753	NS	0.510	0.006	0.930	NS
						
Hyaluronic acid	1.347	0.001	1.228	0.037	1.102	NS
Interleukin-6	1.199	0.024	1.232	0.027	1.052	NS
Interleukin-8	0.880	NS	0.970	NS	0.802	0.042
						
Matrix metalloproteinase-3	1.240	0.026	1.181	NS	1.087	NS
Myeloperoxidase	0.821	NS	0.934	NS	0.694	0.020
*Change from baseline to week 4*						
α_1_-Antitrypsin	0.505	NS	0.298	0.035	0.301	0.033
Apolipoprotein C3	2.191	0.014	1.681	NS	1.741	NS
Basic fibroblast growth factor	0.353	0.007	0.484	0.042	0.424	0.012
Carcinoembryonic antigen	2.674	NS	1.89	NS	3.891	0.020
C-reactive protein	0.714	<0.001	0.652	<0.001	0.728	0.002
ENRAGE (S100A12)	0.642	0.005	0.568	0.001	0.767	NS
Haptoglobin	0.497	0.004	0.417	<0.001	0.474	0.008
Hyaluronic acid	0.513	<0.001	0.514	<0.001	0.699	0.017
Interleukin-6	0.623	<0.001	0.665	<0.001	0.733	0.003
Interleukin-16	0.371	0.007	0.234	<0.001	0.422	0.029
Intracellular adhesion molecule-1	0.654	NS	0.673	NS	0.468	0.030
Lipoprotein (a)	0.727	NS	0.460	0.045	0.748	NS
Matrix metalloproteinase-3	0.539	0.001	0.570	0.005	0.673	0.043
Macrophage inflammatory protein 1-α	0.687	NS	0.454	0.041	0.903	NS
Serum amyloid P	0.628	NS	0.304	0.015	0.346	0.025
Stem cell factor	2.239	0.028	1.521	NS	1.383	NS
Tissue inhibitor of metalloproteinases-1	0.595	NS	0.264	0.035	0.603	NS
Vascular endothelial growth factor	0.419	0.009	0.326	0.004	0.307	0.002

Only biomarkers are shown for which *P *< 0.05 for associations with ACR 20 or ACR 50 responses at each point. ACR, American College of Rheumatology; DAS, disease activity score; OR, odds ratio; MTX, methotrexate; NS, not significant.

In the test dataset, baseline levels of apolipoprotein C3, hyaluronic acid, IL-6, IL-8, MMP-3, and myeloperoxidase were associated with ACR 20, ACR 50, and DAS 28 responses at week 14. An evaluation of biomarker changes from baseline to week 4 yielded a set of markers similar to that identified in the training dataset (including α_1_-antitrypsin, apolipoprotein C3, bFGF, carcinoembryonic antigen, CRP, ENRAGE, haptoglobin, hyaluronic acid, IL-6, IL-16, ICAM-1, lipoprotein (a), MMP-3, MIP-1α, serum amyloid P, stem cell factor, TIMP-1, and VEGF) that were associated with clinical response at week 14 (Table 2).

### Comparisons of predictive values of CRP versus combination of markers

With a logistic regression analysis, the best combination of markers (based on change from baseline to week 4) that were associated with ACR 20 and ACR 50 responses at week 14 is listed in Table 3. This combination includes seven markers, several of which have not been shown to be associated with RA or response to anti-TNF-α treatment. The change from baseline to week 4 in hyaluronic acid and apolipoprotein C3 were the strongest predictors of ACR 20 response at week 14, followed by baseline levels of rheumatoid factor. Only three of these markers (hyaluronic acid, apolipoprotein C3, and IL-16) plus haptoglobin, swollen and tender joint count at baseline, and anti-CCP antibodies were identified as important factors in the prediction of ACR 50 response. Despite being included as one part of the ACR-response criteria, CRP was important for ACR 20 response prediction, but not for ACR 50 response. The NPV and PPV values for CRP alone were lower than the best combination of markers for prediction of ACR 20 and ACR 50 responses, indicating that it is possible for a panel of markers to outperform CRP in monitoring the responsiveness of patients to anti-TNF-α treatment. ESR analyses were slightly less predictive of ACR 20 or ACR 50 responses than was CRP (data not shown).

**Table 3:** Logistic regression predictive models results by using changes in biomarker levels from baseline to week 4 and clinical response at week 14. Model included training and test datasets combined

ACR 20			
*CRP-only model*^a ^*(n = 308)*			

True/Predicted	NR	R	Accuracy
NR	35	115	Specificity = 23%
R	16	142	Sensitivity = 90%
Predictive value	NPV = 68%	PPV = 55%	
Threshold = -0.35			
			

*Biomarker model*^a ^*(n = 308)*			

True/Predicted	NR	R	Accuracy
NR	62	88	Specificity = 41%
R	16	142	Sensitivity = 90%
Predictive value	NPV = 79%	PPV = 61%	

Threshold = -0.35			
			

Predictors from biomarker model^b^	Weight	Odds ratio	Multivariate *P *value

Hyaluronic acid	0.60	1.82	0.0001
Apolipoprotein C3	-0.98	0.38	0.003
Rheumatoid factor positive at baseline	-0.91	0.4	0.014
Basic fibroblast growth factor	0.72	2.05	0.023
Interleukin-16	0.83	2.29	0.031
Interleukin-6 serum	0.21	1.23	0.049
C-reactive protein	0.17	1.19	0.062

			
**ACR 50**			

*CRP-only model*^a ^*(n = 308)*			

True/Predicted	NR	R	Accuracy
NR	108	116	Specificity = 48%
R	17	67	Sensitivity = 80%
Predictive Value	NPV = 86%	PPV = 36%	
Threshold = -1.3			
			

*Biomarker model*^a ^*(n = 308)*			

True/Predicted	NR	R	Accuracy
NR	137	87	Specificity = 61%
R	17	67	Sensitivity = 80%
Predictive value	NPV = 89%	PPV = 44%	
Threshold = -1.2			
			

Predictors from biomarker model^b^	Weight	Odds ratio	Multivariate *P *value

Hyaluronic acid	0.53	1.70	0.001
Haptoglobin	0.79	2.2	0.001
Apolipoprotein C3	-0.94	0.39	0.007
Interleukin-16	1	2.72	0.014
STJC at baseline	0.17	1.19	0.015
Anti-CCP antibodies	-0.82	0.44	0.044

^a^All biomarker values were log transformed before inclusion in the models. Model accuracy (sensitivity, specificity, NPV, PPV) was estimated by using cross validation. ^b^Weights are the coefficients in the logistic regression model. Odds ratios are the exponential of the weights. Multivariate *P *values are the *P *values when all of the terms are included in the model. ACR, American College of Rheumatology; anti-CCP, cyclic citrullinated peptide; CRP, C-reactive protein; NPV, negative predictive value; NR, nonresponder; PPV, positive predictive value; R, responder; STJC, swollen and tender joint count.

### Associations between biomarker levels and health-related quality-of-life outcomes

We examined the associations between biomarker levels and patient reported measures of health-related quality of life (SF-36) and fatigue (FACIT-F). We previously showed that RA patients treated with golimumab with or without MTX showed significantly greater improvement from baseline in SF-36 physical and mental component scores (PCS and MCS) and FACIT-F scores compared with placebo plus MTX at week 14 [15]. In the current study, although several significant associations were found between selected biomarker levels and FACIT-F and SF-36 scores in the training dataset (Table 4), most of these findings were not reproduced in the test dataset, possibly because the original sample size was very limited. In the combined dataset, associations between lower baseline levels of α-antitrypsin, ICAM-1, TIMP-1, and von Willebrand factor and improvement in PCS at week 14 were observed. Low levels of ENRAGE at baseline were also associated with improvement in FACIT-F scores at week 14. Decreases from baseline to week 4 in CRP, ENRAGE, and IL-6 levels were associated with improvement in PCS, and decreases in MMP-3 levels were associated with improvement in MCS.

**Table 4:** Odds ratios for associations between biomarker levels (baseline and changes from baseline to week 4) and outcome measures of fatigue (FACIT-F) and health-related quality of life (SF-36) at week 14 for the training and test golimumab/golimumab + MTX datasets

	FACIT-F		SF-36 PCS		SF-36 MCS	
	
	OR	*P*	OR	*P*	OR	*P*
**Training subset**						
*Baseline*						
von Willebrand factor	0.380	0.021	0.427	0.039	0.370	0.019
*Change from baseline to week 4*						
ENRAGE (S100A12)	0.578	0.053	0.549	0.044	0.603	0.070
Matrix metalloproteinase-3	0.529	0.041	0.621	0.122	1.000	0.999
**Test subset**						
*Baseline*						
Matrix metalloproteinase-3	1.233	0.035	0.922	0.409	0.972	0.775
Tissue inhibitor of metalloproteinase-1	0.925	0.843	0.375	0.017	0.798	0.567
*Change from baseline to week 4*						
C-reactive protein	0.879	0.122	0.772	0.003	0.833	0.033
Matrix metalloproteinase-3	0.893	0.522	0.811	0.248	0.615	0.010
**All data combined**						
*Baseline*						
α_1_-antitrypsin	1.206	0.479	0.567	0.037	1.230	0.436
Intracellular adhesion molecule-1	1.166	0.484	0.611	0.046	1.451	0.117
Tissue inhibitor of metalloprotienases-1	0.833	0.583	0.342	0.002	0.843	0.610
von Willebrand factor	0.949	0.775	0.628	0.017	0.760	0.146
*Change from baseline to week 4*						
C-reactive protein	0.910	0.176	0.831	0.011	0.940	0.370
ENRAGE (S100A12)	0.761	0.040	0.710	0.011	0.821	0.135
Interleukin-6	0.953	0.543	0.846	0.039	0.965	0.651
Matrix metalloprotienase-3	0.810	0.173	0.784	0.121	0.669	0.013

Odds ratios show association between biomarker levels (baseline or changes from baseline to week 4) and whether FACIT-F, SF-36 mental component summary score, or SF-36 physical component summary score was above or below the median. Only biomarkers for which at least one association was <0.05 are shown. FACIT-F, Functional Assessment of Chronic Illness Therapy -Fatigue; SF-36, 36-question Short Form Survey; OR, odds ratio; MCS, mental component summary score; PCS, physical component summary score.

## Discussion

In this study, we evaluated an array of 107 serum proteins and showed that golimumab treatment with or without MTX is effective in modulating certain acute phase reactants (CRP, α_1_-antitrypsin, von Willebrand factor, and haptoglobin), inflammatory markers (IL-6 and ENRAGE), and other selected proteins (bFGF, apoliprotein C3, and serum amyloid P) in two separate datasets from the same study of patients with inadequate responders to MTX. The robustness of the analyses can be attributed to the minimal variability observed between the different platforms used for testing levels of these proteins (Rules-Based Medicine, Luminex, and ELISA). Thus, this provided us with a high level of confidence in the reproducibility of the changes and associations observed.

In the golimumab-treated patients, ACR 20 and ACR 50 responders displayed a distinct serum protein signature as compared with nonresponders, which confirms the importance of the significant markers (IL-6, CRP, haptoglobin, IL-16, VEGF, bFGF, ENRAGE, hyaluronic acid, and MMP-3) in the rheumatologic disease processes. Further, strong associations were shown between the levels of some of these markers (at baseline and changes from baseline to week 4) and response to multiple clinical measures (ACR 20, ACR 50, and DAS 28) after 14 weeks of treatment. In this study, we were also able to show significant associations between changes in measures of patient reported outcomes (SF-36 and FACIT-F) and the changed levels of some of the markers in patients treated with golimumab. The results revealed a link between improvements in disease markers of inflammation and improvement in patient reported outcome measures.

Good evidence exists for the role of several of these markers in perpetuating disease in RA patients. Markers such as CRP [16], haptoglobin, apolipoprotein C3 [17], and ENRAGE [18] have been associated with the early, acute phase responses that occur in RA. Additionally, Charles-Schoeman and colleagues [17] showed that patients with RA with proinflammatory high-density lipoprotein (HDL) exhibit increases in haptoglobin and apolipoprotein levels as compared with patients with anti-inflammatory HDL.

In contrast to the acute-phase response markers, IL-6, IL-16, fibroblast growth factor (FGF), VEGF, hyaluronic acid, and MMP-3 have all been linked to the structural changes that occur during disease progression in RA. IL-6 levels in synovial fluid have previously been associated with local joint-activity score [19] in addition to swollen/tender joint counts in RA [20]. Recent data by Warstat *et al*. [21] showed that synovial fibroblasts from RA patients stimulated with transforming growth factor-β1 on laminin 111 exhibit increases in IL-16 gene expression as compared with osteoarthritis synovial fibroblasts. FGF has been shown to have a role in both matrix synthesis and degradation. Elevated levels of FGF in cartilage have been associated with not only arthritic disease leading to joint destruction [22], but also in mediating cartilage regeneration [23]. VEGF expression is induced by hypoxic conditions that occur in the RA joint, and osteoclast expression of VEGF is mediated through NF-κB [24]. Further, fibroblast-like synoviocytes under hypoxic conditions exhibit elevated MMP-3 levels [25], and a polymorphism in the MMP-3 gene has been shown to be associated with radiographic progression [26]. Elevated levels of hyaluronic acid have been observed in serum from RA patients, and this correlated with clinical parameters [27,28]. Together, this information provides support for the role of these markers in RA disease pathogenesis.

The combination of markers in our model (based on early changes from baseline to week 4 values) was superior to CRP alone in predicting response to golimumab treatment. These markers included CRP, hyaluronic acid, bFGF, apolipoprotein C3, rheumatoid factor, IL-16, and IL-6; however, the NPV and PPV values were not indicative of a strong prediction of response. Results published by Hueber *et al*. [29] identified a 24-serum marker signature that was also weakly predictive of response to etanercept treatment. Further, with the exception of IL-6, none of these markers overlapped with the markers that we have identified in the current study.

Several limitations existed in the study. First, the number of patients in the training dataset was small, making it difficult to reproduce findings in the larger test dataset. Also, it would have been useful to have an earlier collection of samples prior to week 4, enabling identification of earlier, and perhaps stronger, associations between biomarker levels and clinical measures of response. We also observed very low CRP values in patients, and this made it difficult to show further reductions and correlations. An earlier collection of samples would have led us to a better understanding of the fluctuations of specific markers in response to treatment and in relation to changes in disease activity. Lastly, a stronger analysis might have been possible by including profiling of RNA from peripheral blood mononuclear cells, enabling the evaluation of an even larger number of analytes and the inclusion of other key molecules in the TNF-α pathway [30].

## Conclusions

Clearly, a clinical response to golimumab involves modulation of several RA disease processes, including those involved in the acute and inflammatory phase of disease, as well as downstream aspects relating to bone and cartilage metabolism and destruction. The results of this study from two separate datasets showed strong associations between selected biomarker levels and improvement in a variety of clinical-response measures after treatment with golimumab. Baseline levels of markers were not consistently associated with future response to golimumab therapy. The best set of markers associated with response to golimumab treatment included week-4 changes from baseline; however, even these markers were unable to achieve high enough specificity and sensitivity to be routinely useful predictors. Thus, additional testing of serum and other types of markers from other studies will be needed to identify additional molecules that can either be added to strengthen this panel or be used independently as predictive markers in the management of patients with RA who are treated with anti-TNF-α therapies.

## Abbreviations

ACR: American College of Rheumatology; bFGF: basic fibroblast growth factor; CCP: cyclic citrullinated peptide; CRP: C-reactive protein; COMP: cartilage oligomeric matrix protein; DAS: Disease Activity Score; ELISA: enzyme-linked immunosrobent assay; ENA: epithelium-derived neutrophil-activating protein; ESR: erythrocyte sedimentation rate; FACIT-F: Functional Assessment of Chronic Illness Therapy -Fatigue; FDR: false discovery rate; FGF: fibroblast growth factor; ICAM: intracellular adhesion molecule; IL: interleukin; MCP: monocyte chemotactic protein; MDC: monocyte/macrophage-derived chemokine or CCR-4; MCS: mental component score; MIP: macrophage inflammatory protein; MMP: matrix metalloproteinase; MTX: methotrexate; NF: nuclear factor; NPV: negative predictive value; P1NP: N-terminal propeptide of type 1 procollagen; PAI: plasminogen activator inhibitor; PCS: physical component score; PPV: positive predictive value; RA: rheumatoid arthritis; SF-36: 36-question Short Form Survey; SGOT: serum glutamic oxaloacetic transaminase; SHBG: sex hormone-binding globulin; TIMP: tissue inhibitor of metalloproteinase; TNF-α tumor necrosis factor-α VEGF: vascular endothelial growth factor.

## Competing interests

EK, MG, and LK (or their institutions) have received research grants from Centocor and/or Schering-Plough. EK, LK, and ME have received consulting fees from Centocor and/or Schering-Plough. SV, MUR, EH, MM, JB, and CW were Johnson & Johnson employees and owned stock and/or stock options at the time this work was conducted.

Centocor Research Development, Inc., is a wholly owned subsidiary of Johnson & Johnson, Inc. Johnson & Johnson and Schering-Plough (now Merck & Co.) own the patent for SIMPONI (golimumab).

## Authors' contributions

EK, MG, LK, EH, MM, and MUR designed the study and oversaw the study conduct and data acquisition. SV and CW designed and conducted the biomarker analyses. ME conducted the statistical analysis of the biomarker data. SV and CW drafted the manuscript with the assistance of a medical writer (see Acknowledgments) who did not meet the criteria for authorship. All authors reviewed the manuscript, revised it critically, and approved the final version for submission.
